# Combining a small urinary catheter with an endo mini-retractor: a novel technique for laparoscopic selective vascular control

**DOI:** 10.1093/jscr/rjac227

**Published:** 2022-07-19

**Authors:** Vor Luvira, Apichaya Junyavoraluk, Theerawee Tipwaratorn

**Affiliations:** Department of Surgery, Faculty of Medicine, Khon Kaen University, Khon Kaen, Thailand; Department of Surgery, Faculty of Medicine, Khon Kaen University, Khon Kaen, Thailand; Department of Surgery, Faculty of Medicine, Khon Kaen University, Khon Kaen, Thailand

## Abstract

The Pringle maneuver using a urinary catheter has been described and proven to be one of the best methods for laparoscopic intracorporeal vascular control. Selective vascular control provides many advantages over total inflow occlusion by the Pringle maneuver. However, laparoscopic selective vascular control by urinary catheter has not been described. We, herein, describe laparoscopic selective vascular control using a combined small urinary catheter with an endo mini-retractor, which inserted in a new way, named ‘The J-shaped Loop’. This method makes the instrument more compact so we can selectively control the vascular pedicle. When selective vascular occlusion is desired, the loop is tightened and the tension is held just by the catheter itself. This novel technique is simple, safe, effective and reproducible, and, therefore, can be used as a good alternative to other intracorporeal techniques for laparoscopic liver resection.

## INTRODUCTION

Vascular control during laparoscopic liver resection is an essential step to minimize blood loss and improve surgical outcomes. There are many techniques of laparoscopic vascular control [1]. Of these, the Pringle maneuver using a urinary catheter has been described and proved to be an effective method with many advantages, including rapid clamping/ unclamping time, no need for an additional port, is less traumatic, has a lower risk of incomplete occlusion, and has no risk of gas leak during the operation [2, 3].

Selective vascular inflow occlusion, instead of interrupting all inflows, has been proved to reduce liver damage [4], and is an aid in identification of the segment border by demarcation. However, laparoscopic selective vascular control by urinary catheter has not been described, after extensive search for this method in medical databases (i.e. Medline, Scopus and EMBASE) and other multimedia. We therefore developed a combined small urinary catheter with Endo mini-retractor as a novel technique for laparoscopic selective vascular control. This article describes our technique for laparoscopic selective vascular control using a urinary catheter, named The J-shaped Loop (TJL).

## TECHNIQUE

Laparoscopic vascular inflow occlusion by total clamping of the hepatoduodenal ligament using a urinary catheter has been routinely used in General 4 Division, Department of Surgery, Khon Kaen University since 2019. We later modified this to ‘The J-shaped Loop’ (TJL) by a combination of a 10 Fr. urinary catheter with an endo mini-retractor in 2021. We encircled the hepatoduodenal ligament using a 16 Fr. Urinary catheter in all cases of laparoscopic liver resection in order to promptly control unexpected bleeding when needed. We performed selective extra-Glissonian pedicle control at the same side of the planned resection.

**Figure 1:**
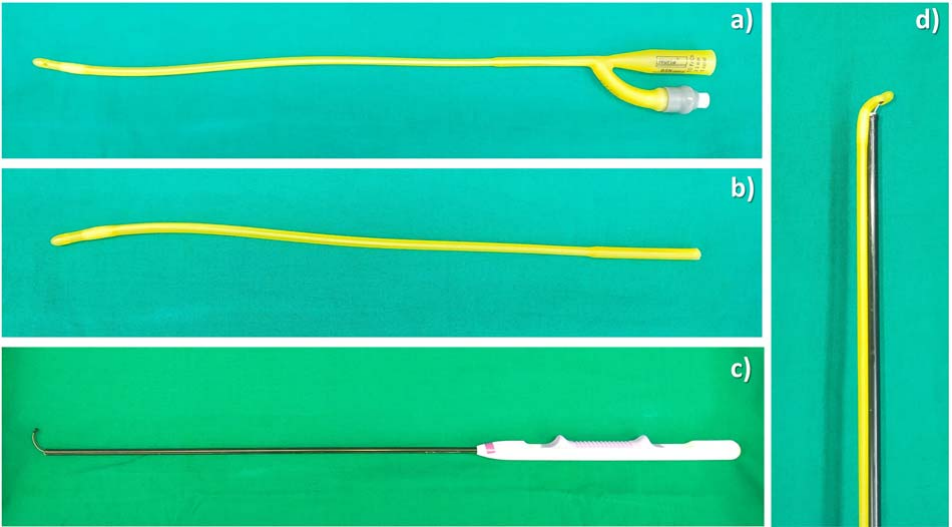
Instruments for creation of TJL. (**a**) A 10-Fr urinary catheter; (**b**) an end-cut 10 Fr urinary catheter; (**c**) endo mini-retractor; (**d**) The J-shaped Loop.

**Figure 2:**
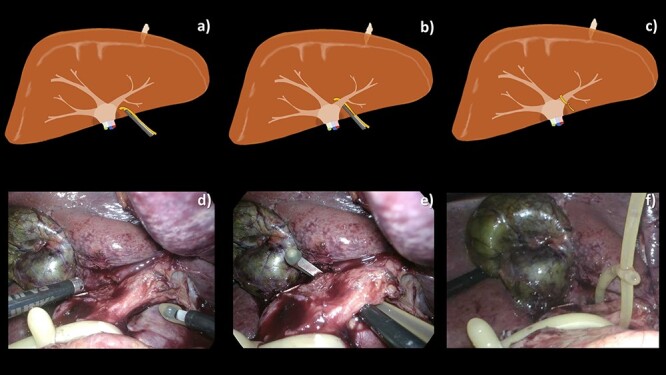
A schematic illustration (**a**–**c**) and intraoperative images (**d**–**f**) of our operative technique. (a, d) TJL was introduced into the abdominal cavity via an 11 mm port. (b, e) Extra-Glissonian encirclement of the left pedicle. (c, f) The loop was created completely by passing the tail of the catheter through both side-holes of tip of the catheter.

The patient was lying in the French position. Ports were placed; 4–5 ports (11 mm × 2, 5 mm × 3) were usually required in all operations. Sometimes, an additional 1–2 ports were required for a safer procedure. Pneumoperitoneum was created, up to 15 mmHg. The liver was mobilized and resectability was evaluated. The hepatoduodenal ligament was identified and encircled using a 16 Fr urinary catheter. The Glissonian pedicle of the planned resection liver was gently dissected for selective vascular control. TJL was created, using an end-cut 10-Fr. Urinary catheter (Fig. 1a and b) and endo mini-retractor (Fig. 1c). The tip of the retractor was inserted, via the one of the side-holes of the urinary catheter, pointing toward the tip of the urinary catheter (Fig. 1d). ‘TJL’ was inserted to the abdominal cavity via the 11-mm port (Fig. 2a), and then encircled the previously dissected pedicle (Fig. 2b). The tip of the urinary catheter was grasped from another side of the pedicle. The dissector was used for making the loop, by passing the dissector through both side-holes at the tip of the urinary catheter, then, holding the cut end of the catheter, pulling back through the holes (Fig. 2c). The loop was later trimmed. When selective vascular occlusion was desired, the loop was tightened and the tension was held just by the catheter itself. (Supplementary Material, Video)

**Figure 3:**
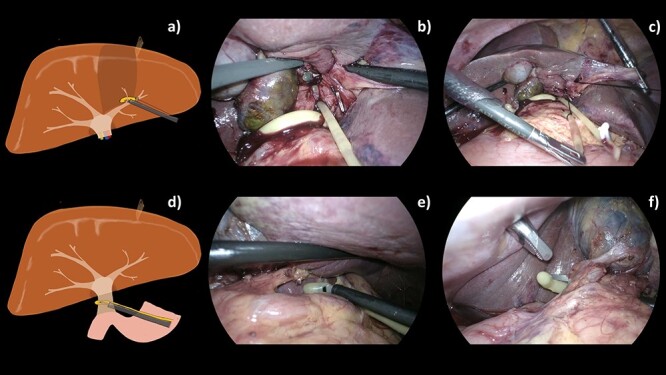
A schematic illustration (**a**, **d**) and intraoperative images (**b**–**c**, **e**, **f**) of our operative techniques for selective control of the segmental pedicle and hepatoduodenal ligament (HDL). (a) Selective extra-Glissonian control of S4 pedicle. (b) Combination of TJL and 2–0 Silk was introduced into abdominal cavity. (c) S4 pedicle was controlled by 2–0 Silk. (d) Encircling of hepatoduodenal ligament. (e, f) Combined 16-Fr urinary catheter with endo mini-retractor for control of the hepatoduodenal ligament.

This technique, combining a urinary catheter with an endo mini-retractor, can be used for controlling the segmental pedicle (Fig. 3a–c) and hepatoduodenal ligament; the Pringle maneuver (Fig. 3d–f).

## DISCUSSION

**Figure 4:**
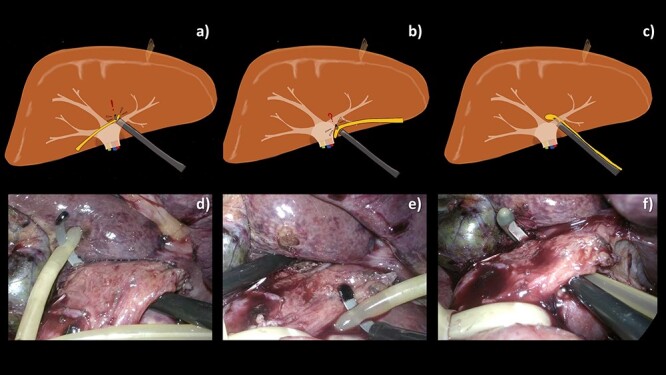
Comparative schematic illustrations (**a**–**c**) and intraoperative images (**d**–**f**) of various techniques attempted for selective encirclement of pedicle by a 10-Fr urinary catheter. (a, d) Feeding the tube to the endo mini-retractor after encircling the pedicle by the retractor making a difficulty in retrieval of the catheter back. (b, e) Piercing the endo mini-retractor through both holes at the tip of the catheter making the catheter prone to slip along the curve of the retractor tip. (c, f) With our technique, the catheter is easily encircling the pedicle.

We have proposed a novel simplified technique to selectively encircle the vascular pedicle using a urinary catheter. We believe that intracorporeal vascular control using a urinary catheter is one of the best methods, and selective vascular control shows many advantages over the Pringle maneuver [4]. We therefore attempted to achieve selective vascular control using a urinary catheter. We had tried several methods and failed to selectively encircle the pedicle because of the limited space between the vascular pedicle and liver parenchyma (Fig. 4). Finally, we found that insertion of the retractor tip via one of the side-holes of the urinary catheter, pointing towards the tip of the catheter, made the instrument more compact, so we can control the catheter more easily.

**Figure 5:**
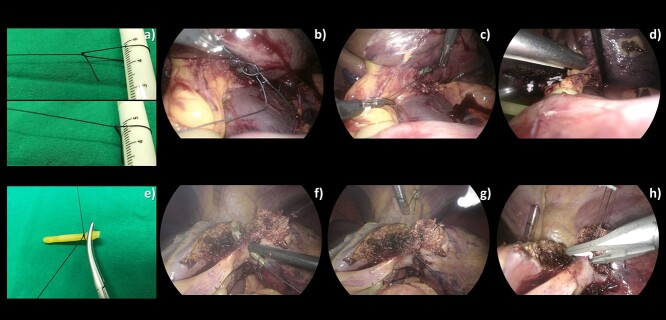
A comparison of intraoperative images of a traditional (**a**–**d**) and our technique (**e**–**h**) for encircling Glissonian pedicle before stapling or clipping. (a) An extracorporeal adjustable knot was created. (b) The loop was introduced into the abdominal cavity and worn around the neck of the endo mini-retractor. (c) The loop was tightened around the retractor neck. (d) The pedicle was encircled by 2–0 Silk before being stapled. (e) 2–0 Silk was tied to end of the ripped catheter using a basic square knot. (f, g) The combined 10-Fr urinary catheter with endo mini-retractor was used to encircle the pedicle. (h) The pedicle was clipped after being controlled by 2–0 Silk.

For laparoscopic selective Glissonian pedicle approach, owing to movement limitations of basic instruments, some special instruments are required, for instance, Goldfinger dissector (Johnson & Johnson, Le Locle, Switzerland), Endo Retract Maxi 10 mm (Covidien, Tokyo, Japan) and Endo Mini-Retract 5 mm (Covidien), etc. [5]. Among these, endo mini-retractor is the most available one and, moreover, its size is the most appropriate for encircling the second and third order pedicles. Despite an aid of these special instruments, the dissection has to be performed blindly at the posterior side, leads to some injuries and bleeding [5]. Our technique could solve this issue, as it simply provides non-traumatic tunneling, because the softness of the urinary catheter makes the tip of the retractor blunt. In addition, when the pedicle need to be encircled by suture materials before being either clipped, or stapled, the surgeons traditionally tie the sutures directly to the tip of an endo-retractor using advanced adjustable knots, for example, Meltzer’s Knot, Roeder’s Knot, Tayside Knot etc., those require training of tying skill (Fig. 5 a–d). Our method, combining endo mini-retractor and urinary catheter, allows surgeon to tie suture material to the end of the urinary catheter using only basic knots (Fig. 5 e–h). Covering the tip of surgical instruments by a urinary catheter can be implemented with any surgical instruments with an appropriate curve. In open surgery, we also use this technique when we need to either encircle or control the vascular pedicle, by combining a urinary catheter with a right-angle instrument.

TJL can be prepared either extracorporeally (with the requirement of an 11-mm port) or intracorporeally (no requirement of 11-mm port, but more difficult). The reason we chose the 10-Fr catheter was because the 10-Fr is not only smaller than the 12-Fr, but shorter, making it more suitable for laparoscopic surgery. Moreover, this size fits the tip of the retractor and provides an appropriate tightness to occlude pedicle inflow without the requirement for a Hem-o-lock clip or Metallic clip. We also use this technique for encircling the hepatoduodenal ligament (the intracorporeal Pringle maneuver) by a 16-Fr urinary catheter, which is big enough to hold the tightness to occlude all hepatic inflow without the requirement of any clip. Moreover, this technique can be performed even in the presence of adhesion around the hepatoduodenal ligament.

To the best of our knowledge, this is the first paper describing selective pedicle control using a urinary catheter. Moreover, this technique, covering the tip of surgical instruments by a urinary catheter, can be further not only applied to encircle other vascular structures of other organs apart from the liver, but also implemented with any surgical instruments other than an endo mini-retractor, either in open or laparoscopic surgery.

## CONCLUSION

Laparoscopic selective vascular control using a combination of a small urinary catheter with an endo mini-retractor is simple, safe, effective and reproducible, and, therefore, can be used as a good alternative to other intracorporeal techniques for laparoscopic liver resection.

## Supplementary Material

TJL_1_1_Voice_rjac227
